# Supplementary material for: Temporal artery temperature measurements versus bladder temperature in critically ill patients, a prospective observational study
Source: PLoS One. 2020 Nov 6;15(11):e0241846. doi: 10.1371/journal.pone.0241846 (PMC7647096; doi:10.1371/journal.pone.0241846)
Supplement: S1 Table — TAT1: temporal artery measurement by observer 1; TAT2: temporal artery measurement by observer 2. (DOCX) [file pone.0241846.s001.docx]

**S1 Table. Deviation of temperature measurements using different acceptable margins**

| Acceptable margin (°C) | % deviating from the acceptable margin (TAT_1_) | % deviating from the acceptable margin (TAT_2_) |
| --- | --- | --- |
| 0.1°C | 91.1 | 92.2 |
| 0.2°C | 72.2 | 71.1 |
| 0.3°C | 55.6 | 51.1 |
| 0.4°C | 37.8 | 44.4 |
| 0.5°C | 35.6 | 42.2 |
| 0.6°C | 26.7 | 28.9 |
| 0.7°C | 23.3 | 18.9 |
| 0.8°C | 21.1 | 14.4 |
| 0.9°C | 16.7 | 8.9 |
| 1.0°C | 14.4 | 8.9 |
