# Supplementary material for: Temporal artery temperature measurements versus bladder temperature in critically ill patients, a prospective observational study
Source: PLoS One. 2020 Nov 6;15(11):e0241846. doi: 10.1371/journal.pone.0241846 (PMC7647096; doi:10.1371/journal.pone.0241846)
Supplement: Attachment — Submitted filename: Rebuttal PLOS one.docx [file pone.0241846.s003.docx]

Editor:

1) Were the examiners blinded (TAT 1 and TAT 2) to each other and to the bladder temperature?

*Author response: Dear Editor, thank you very much for your comments. The examiners were not blinded to each other nor to the bladder temperature. We have now added this as a limitation in the discussion.*

2) What is “moisture” included alongside of vasopressors and heating in table?

*Author response: We have changed the phrase ‘moisture’ to ‘moist skin’ as this was indeed unclear. With moist skin we indicate a sweaty skin, signs which may be present in sepsis and fever.*

3) Did you compare the absolute values of the differences between the two methods of temperature evaluation? It seems the mean is close to zero but the SD are quite large. And the absolute magnitude of the deviation is at least as meaningful as the direction (too hot / too cold) of the deviation. If not, I would like to see it run as a post-hoc analysis.

*Author response: Thank you for pointing at this. We have compared this in the results section, but this was not clear enough. We have now added an extra sentence to clarify this finding.*

Journal Requirements:

1. Please ensure that your manuscript meets PLOS ONE's style requirements, including those for file naming. The PLOS ONE style templates can be found at

https://journals.plos.org/plosone/s/file?id=wjVg/PLOSOne_formatting_sample_main_body.pdf and

https://journals.plos.org/plosone/s/file?id=ba62/PLOSOne_formatting_sample_title_authors_affiliations.pdf

2. Please describe in your methods section how capacity to consent was determined for the participants in this study.

3. Please provide additional details regarding participant consent. In the ethics statement in the Methods and online submission information, please ensure that you have specified what type you obtained (for instance, written or verbal, and if verbal, how it was documented and witnessed). If your study included minors, state whether you obtained consent from parents or guardians. If the need for consent was waived by the ethics committee, please include this information.

*Author response: We have amended the manuscript and have added all above mentioned points to the manuscript.*

4. We note that you have indicated that data from this study are available upon request. PLOS only allows data to be available upon request if there are legal or ethical restrictions on sharing data publicly. For information on unacceptable data access restrictions, please see http://journals.plos.org/plosone/s/data-availability#loc-unacceptable-data-access-restrictions.

a) If there are ethical or legal restrictions on sharing a de-identified data set, please explain them in detail (e.g., data contain potentially identifying or sensitive patient information) and who has imposed them (e.g., an ethics committee). Please also provide contact information for a data access committee, ethics committee, or other institutional body to which data requests may be sent.

b) If there are no restrictions, please upload the minimal anonymized data set necessary to replicate your study findings as either Supporting Information files or to a stable, public repository and provide us with the relevant URLs, DOIs, or accession numbers. Please see http://www.bmj.com/content/340/bmj.c181.long for guidelines on how to de-identify and prepare clinical data for publication. For a list of acceptable repositories, please see http://journals.plos.org/plosone/s/data-availability#loc-recommended-repositories.

*Author response: There are no ethical or legal restrictions on sharing the de-identified data set. We have uploaded a minimal and anonymized data set as Supporting Information file.*

Reviewers' comments:

Comments to the Author

Reviewer #1: Peer Review for manuscript titled: Temporal auricular temperature measurements versus bladder temperature in critically ill patients, a prospective observational study

Thank you for submitting this manuscript for the method-comparison study aimed to evaluate the degree and limits of agreement between temporal artery temperature (TAT) and bladder temperature (BT) measurements. This study found good agreement between these thermometry methods with a mean difference (bias) of 0.12° C within the a priori set clinically meaningful threshold of < 0.5° C and the poor limit of agreement (precision) with a SD ± 0.61° C (95% CI -1.08°C, 1.32°C). This was a well-designed and executed study that addresses a gap in the method-comparison literature regarding thermometry methods used in hospitalized and critically ill patients, especially when non-invasive methods are preferred to reduce device-associated risk of harm to patients. I have primarily minor recommendations for the authors regarding the manuscript regarding clarifications of the methods, design, and result figures requiring attention prior to acceptance for publication.

*Author response: Thank you for your comments and reviewing this manuscript.*

1. Terminology/definitions throughout the manuscript: 1) The title and a few times in the manuscript, temporal auricular temperature or “auricular” is used (title, line 116, figure 2). I am not sure if this is a typo or misuse of the name of the thermometry method. Recommend using what is used in the literature, “Temporal Artery Temperature” when TAT is referred to. 2) TAT is obtained via a non-invasive method and is a peripheral temperature. Urinary bladder temperatures are obtained via an invasive method and is considered a core temperature (very good agreement with the gold standard pulmonary artery blood temperature obtained from a PA catheter with a thermistor). It is not customary to refer to BTs as semi-invasive than PA catheter rather these are either invasive or non-invasive.

*Author response: Thank you very much for pointing at this inconsistency. We have now changed this throughout the whole manuscript.*

2. Study design: this is a method-comparison study design. You reference Bland & Altman (1999) –they also have more current design/analysis publications on method-comparison studies.

*Author response: Indeed, thank you for pointing this out. We will update our references.*

3. Line 86: Recommend adding a statement with the rationale for conducting the subgroup analysis comparing vasopressor to non-vasopressor use.

*Author response: We have added a sentence to line 79 to clarify the rationale for conducting the subgroup analysis.*

4. Methods: 1) Was TBI considered an exclusion criteria? It is not listed in the methods section, yet was referred to as a reason for exclusion in the Results section. 2) Measurement: Excellent that you were able to include 2 observers to test for inter-rater reliability issues. Check for a typo in line 113 (intra-rater vs inter-rater). 3) Consider adding a description of how the TAT is obtained since there are more than one procedure in the literature (temporal artery forehead scan vs temporal artery forehead along face to behind ear scan techniques –see Lawson L, Bridges EJ, et al (2007) and Carroll E, et al (2004).

*Author response: 1) The accessibility of the places for measurement was mentioned in the methods section (line 99), which can be inaccessible due to TBI. 2) We have now changed this. 3) We have added a sentence (line 112-114) to clarify this in the methods section under measurement procedure.*

5. Results: 1) Although you collected 2 sets of data per patient (TAT1 and TAT2) for the inter-rater reliability test, it is unclear if there is a need to present the data in Figure 2 (scatter plots of correlations –which is not relevant to inter-rater reliability). The correlation result of r = 0.94 analyzed with the Pearson Correlation Coefficient demonstrates excellent inter-rater reliability. 2) Figure 3: Bland Altman plots: Although described in the legion, it would be preferred to have the LOA values in the figure aside the dashed lines.

*Author response: 1) We believe that such scatter plots may aid the reader with interpreting our data and results. We agree however with you comment and we leave it up to the editor whether we will remove this figure. 2) We have now added the LOA values to the Bland Altman plots.*

6. Discussion: 1) Line 217-220: typo and consider use of standard reference for “mean difference” (bias) as the quantified degree of agreement and “limits of agreement” (precision) as SD and the 95% LOAs. 2) Line 240 –this statement re: “…although this was not statistically significant” was not clear to me. 3) Line 266-270: recommend using hypothermic and febrile range temperatures. Want to not confuse hyperthermic conditions with fever/febrile conditions.

*Author response: 1) We have changed this in the discussion section (line 242-243). 2) This was indeed not clear; we have now removed this sentence from the manuscript. 3) We have changed this in the manuscript.*

7. References: Recommend a review of literature for more current references

*Author response: We have added and updated some references.*

8. General grammar/typo/etc:

a. Line 71-72: “shock resistant” is not clear –is this a shock state of the patient or a device characteristic?

b. Line 144: clinically acceptable

c. Line 167 –I believe you mean perspiring rather than transpiring.

d. Line 185-186: reword this important result statement to ensure that it is clear that the proportion of measurement differences that were ≥ 0.5°C was 36%. Same recommendation for Line 190-191.

*Author response: We have changed all grammar and typo’s according to the suggestions.*
